# Red Flags, Geography, Exam Scores, and Other Factors Used by Program Directors in Determining Which Applicants Are Offered an Interview for Anesthesiology Residency

**DOI:** 10.7759/cureus.11550

**Published:** 2020-11-18

**Authors:** Rafael Vinagre, Pedro Tanaka, Yoon Soo Park, Alex Macario

**Affiliations:** 1 Anesthesiology, Perioperative and Pain Medicine, Stanford University School of Medicine, Stanford, USA; 2 Medical Education, University of Illinois, Chicago College of Medicine, Chicago, USA

**Keywords:** residency application, step 1 change, step 1 pass/fail, anesthesiology program directors, holistic review residency application, residency and internship, residency interview

## Abstract

Objective

The goal of this study was to measure the most important factors in candidate applications that anesthesiology program directors (PDs) use to decide who to invite for an interview, and how that might change once the United States Medical Licensing Examination (USMLE) Step 1 is only reported as pass/fail.

Design

Based on a literature review, a comprehensive list of 27 factors used by PDs to select candidates for the interview was developed. An anonymous survey link was emailed to PDs of all Accreditation Council for Graduate Medical Education (ACGME) accredited Anesthesiology residencies. The survey asked PDs to rank order the top 10 factors they currently consider for making interview invitation, and then to repeat the rank ordering as if the USMLE Step 1 score was instead reported as pass/fail as will be done beginning in 2022.

Results

Forty-five of 159 (28%) PDs responded, with 82% disagreeing with changing the Step 1 score to pass/fail. 84% consider the Step 1 score (77% for Step 2) moderately or very important for selecting an applicant for an interview. The most frequently mentioned “red flags” were failure of a licensing exam, failure of a medical school course, gaps in education without explanation, and criminal history. 69% of PDs agreed that applicants coming from the medical school affiliated with their program would have an advantage over other applicants. Although, the three factors most commonly ranked in the top 10 in importance were the Step 1 score, followed by letters of recommendation, and then the Medical School Performance Evaluation, variability exists in how PDs ranked factors. For example, of the PDs that had Step 1 in the top 10, 27% had it ranked between the 6th and 10th most important. 9% of PDs did not have Step 1 score in the top 10. Core clinical clerkship grades were one of the top 5 factors by 49% of PDs, yet overall was the 6th most common top 10 factor as 36% of PDs did not have core clerkship grades at all in the top 10. Once Step 1 is reported only as pass/fail, PDs had letters of recommendation, Step 2, and the Medical School Performance Evaluation as the most frequently ranked factors in the top 10. 64% of the PDs supported restricting the number of programs a candidate can apply to, with the majority suggesting a limit of 15 to 20 programs per applicant.

Conclusion

Variability exists among anesthesiology PDs in the key criteria for offering an applicant an interview. Once Step 1 is reported as pass/fail, there will be an increased emphasis on Step 2 scores.

## Introduction

The number of residency applications per student has increased due to the competition to secure an interview invitation [1]. This may lead to programs utilizing screening “filters”, such as the United States Medical Licensing Examination (USMLE) Step 1 score [2,3] because it is a national, standardized and objective measure [4]. In the National Residency Matching Program’s (NRMP) 2018 report [5], 94% of Program Directors (PDs) from all specialties cited the Step 1 score as a factor in choosing which applicants to invite to interview with 64% requiring a minimum target score.

Although the Step 1 score may be the single most important factor [6], the USMLEs were designed as a criterion-referenced assessment where the goal is to determine the test-taker’s performance compared to a predetermined standard minimum knowledge that a physician should possess. When the Step 1 score is used as a screening filter instead, it is improperly being used as a norm-referenced test designed to demonstrate how test-takers perform relative to one another. This score is the only measurement available for all applicants prior to the NRMP’s ranking deadline. Overall, U.S. allopathic seniors who matched to their preferred specialty have mean USMLE Step 1 scores of 234 (SD = 17) well above the minimum passing score of 194 [7].

The reliance on the Step 1 score as a way to determine access to interviews increases medical student’s anxiety, disadvantages underrepresented minorities, and may result in medical schools “teaching to test” [8]. Students dedicate much time for Step 1 preparation because unlike the Scholastic Assessment Test or the Medical College Admission Test, which can be taken multiple times to gain a better score, the USMLE Exams are a one-time exam. Therefore, a poor performance is perceived to negatively influence the ability to enter highly competitive specialties, or train at the most sought after residencies.

In February 2020, the National Board of Medical Examiners (NBME) and the Federation of State Medical Boarders (FSMB) announced that beginning in 2022 the Step 1 score will be reported as pass/fail only. The rationale is to change the “step 1 culture” by reducing the overemphasis on Step 1 performance, while maintaining its main purpose for medical licensure eligibility [9].

The goal of this electronic survey study was to measure the most important factors in candidate applications that anesthesiology PDs use to decide who to interview, and how that might change once Step 1 is only reported as pass/fail.

## Materials and methods

Survey instrument

A literature review was conducted through MEDLINE and PubMed, and along with studies drawn from reference lists of relevant articles, a comprehensive list of the factors used by PDs to select candidates for interview was compiled. These factors (e.g., research experience, letters of recommendation, USMLE performance) were listed in random order in the survey questionnaire. A question on red flags that draw concern on a candidate’s application was included. The survey also addressed characteristics on the application that may indicate that an applicant actually may be less likely to be interested in training in the program and therefore less likely to be offered an interview. The survey obtained basic information about the residency programs, including geographic region, and university or community hospital affiliation. The survey instrument included 16-point Likert scale, multiple-choice, and free-text items, as well as an open-ended final written in comments question (Appendix 1). The initial survey instrument was formally tested with a convenience sample of three PDs and three residents. Iterative changes were made until feedback indicated no more changes were needed.

Sampling and data collection

All the PDs of anesthesiology residency programs accredited by the Accreditation Council for Graduate Medical Education (ACGME) were identified through the list of programs on the public ACGME website. E-mail addresses were obtained via department websites. The e-mails inviting PDs to participate provided a link to an anonymous web-based survey hosted by the online survey tool Qualtrics (https://uit.stanford.edu/service/survey). The survey was open from April 1 to May 30, 2020. To protect participant confidentiality, no identifiable information was collected from the respondents. An email reminder was sent weekly to increase the response rate. The survey design allowed participants to return to previous questions and required all questions to be answered before it could be submitted.

Data analysis

PDs rank ordered the top 10 factors they currently consider for interview invites, and then to answer the same question as if the Step 1 score was instead reported as pass/fail. Responses were summarized by determining the % of PDs that ranked that factor as #1, and the % that ranked it as #2, the % that ranked it #3, and so on and then adding the %s. The outcome of interest was the frequency by which a factor was ranked in the top 10 from most to least important. Survey responses in the form of a 4-point Likert scale (1, strongly disagree; 2, disagree; 3, agree; 4, strongly agree) were analyzed with percentages.

## Results

Of the 159 ACGME anesthesiology residency PDs emailed, four were excluded due to non-functional e-mail addresses, with 45 PDs submitting a completed survey (response rate = 28%). Two-thirds of respondents were from a University Hospital program (Table 1).

**Table 1:** Response rate by geographic region in the United States and program type Shows the response rate in each geographic region and the type of program Program Directors are from. *One respondent classified as other, describing program as “healthcare system with medical school, no University”.

	Response Rate
Region	
Midwest	22%
South	20%
West Coast	27%
East Coast	31%
Program Type	
University Hospital	67%
University Affiliated	20%
Community Hospital	9%
Military Program	2%
Other*	2%

The majority (82%) of respondents were not in favor of the scoring change for Step 1, where 51% strongly disagree, 31% disagree, 16% agree and only 2% strongly agree. When asked about the importance of Step 1 and Step 2 scores for interview decisions, 84% and 77% consider them important, respectively. The level of importance was ranked as not at all important (0% for Step 1 and 7% for Step 2), slightly important (16% for Step 1 and 16% for Step 2), moderately important (35% for Step 1 and 44% for Step 2) and very important (49% for Step 1 and 33% for Step2).

Eighty-four percent consider red flags very important when selecting an applicant for an interview. The four most commonly written in responses for “red flags” that raise concern about the applicant were: failure of USMLE exams, failure of a course or clinical rotation, gaps in education/missing time from school without explanation, and any felonies/any other criminal history (Table 2). 

**Table 2:** Red flags PDs look for when reviewing an application Reflects Program Directors (PDs) opinion of red flags they look for when reviewing an application before inviting an applicant for an interview. Respondents could write in more than one response.

	Number of times mentioned
Failure of USMLE exams/multiple attempts	17
Failure of a course or clinical rotation in medical school	16
Gaps in education/missing time from school without explanation	16
Felonies, Misdemeanor, DUI, or any other criminal history	14
Professionalism issues/negative comments	11
Lukewarm/poor /short/generic letters of recommendation	8
Poor USMLE score	6
Extension of medical school time/not graduating on time	5
Poor comments about interpersonal and communication skills	3
Leave of absence from medical school	3
Poor performance on rotations	3
Any course remediation	3
Repeating courses/failing courses or rotations	3
Negative comments on clerkship evaluations	3
Cheating	2
Ethics lapses	2
Probation of any kind	2
Poorly written personal statement	2
Substance use	2
Disciplinary action	2
Any negative comments in Dean's letter	2
Failure to match previously	1
Low class rank	1
Multiple transfers	1
Low numbers of activities (leadership, research, community service)	1
Inconsistent performance	1
Coming from other specialties	1
Research in other specialties	1

The two most important characteristics related to the applicants’ research experience was having “any research experience” (23%) and “research experience but not necessarily publication” (20%) (Table 3).

**Table 3:** Most important characteristics in research experience that PDs look for Shows the most important characteristics Program Directors (PDs) look for in an application regarding research experience.

Any research experience	23%
Research experience but not necessarily publication	20%
Quality of publications (high impact factor)	18%
Research in anesthesiology	13%
Applicant is first author	11%
Quantity of publications	8%
Basic science research	1%
Applicant is middle author	1%

For letters of recommendation, the “content of the letter” and “how well the letter writer knows the applicant” were most important (Table 4).

**Table 4:** Most important characteristics of letters of recommendation PDs look for Shows the most important characteristics of letters of recommendation Program Directors (PDs) look for when reviewing an application.

Content of the letter	33%
How well the letter writer knows the applicant	30%
Specificity of the letter	18%
From someone in the specialty	9%
From someone you know	8%
From a well-recognized person	2%

The majority of program directors (69%) agreed that applicants coming from the Medical School affiliated with their program would have advantage over other applicants. The applicant being from the same State/Area of the residency program was viewed as moderately or very important by 33% of PDs.

The three factors most commonly ranked in the top 10 (from the given list of 27 possible factors) were the USMLE Step 1 score, followed by letters of recommendation, and the Medical School Performance Evaluation (MSPE) (Table 5).

**Table 5:** Ranking of the importance of factors when selecting an applicant for an interview Shows the current importance of each factor in an application in order for the applicant to be invited for an interview. * Ties with local area, any evidence of overall "grit", consistent performance throughout medical school. MSPE stands for Medical School Performance Evaluation.

Factor	#1	#2	#3	#4	#5	#6	#7	#8	#9	#10	Total
USMLE Step 1	28.9%	15.6%	8.9%	4.4%	6.7%	11.1%	8.9%	2.2%	2.2%	2.2%	91.1%
Letter of recommendation	6.7%	8.9%	2.2%	2.2%	8.9%	13.3%	6.7%	11.1%	8.9%	8.9%	77.8%
Dean's Letter (MSPE)	8.9%	8.9%	6.7%	13.3%	6.7%	0.0%	8.9%	6.7%	8.9%	4.4%	73.3%
USMLE Step 2 CK	2.2%	22.2%	8.9%	6.7%	11.1%	6.7%	6.7%	4.4%	0.0%	0.0%	68.9%
Class rank	6.7%	2.2%	11.1%	11.1%	11.1%	4.4%	8.9%	0.0%	4.4%	8.9%	68.9%
Core clinical clerkship grades	8.9%	11.1%	11.1%	11.1%	6.7%	8.9%	2.2%	0.0%	2.2%	2.2%	64.4%
Personal Statement	2.2%	2.2%	4.4%	6.7%	4.4%	2.2%	6.7%	11.1%	8.9%	8.9%	57.8%
Applicant's medical school	2.2%	4.4%	4.4%	6.7%	2.2%	2.2%	8.9%	6.7%	11.1%	2.2%	51.1%
Grade in Anesthesiology clerkship	0.0%	2.2%	4.4%	11.1%	4.4%	6.7%	4.4%	4.4%	8.9%	2.2%	48.9%
Leadership roles	2.2%	2.2%	2.2%	2.2%	4.4%	8.9%	2.2%	4.4%	8.9%	8.9%	46.7%
Rotation at your department	6.7%	6.7%	0.0%	0.0%	2.2%	6.7%	4.4%	0.0%	4.4%	2.2%	33.3%
Written comments about clinical rotation performance	2.2%	4.4%	4.4%	0.0%	11.1%	0.0%	2.2%	6.7%	0.0%	2.2%	33.3%
Country of medical education	13.3%	2.2%	6.7%	2.2%	2.2%	2.2%	0.0%	0.0%	0.0%	2.2%	31.1%
Alpha Omega Alpha Honor Medical Society	4.4%	0.0%	6.7%	0.0%	2.2%	0.0%	2.2%	6.7%	6.7%	0.0%	28.9%
Performance in preclinical courses	0.0%	2.2%	2.2%	2.2%	2.2%	8.9%	2.2%	2.2%	2.2%	4.4%	28.9%
The number of Anesthesiology electives	0.0%	2.2%	6.7%	4.4%	2.2%	2.2%	2.2%	4.4%	0.0%	4.4%	28.7%
Research Experience in any field	2.2%	0.0%	0.0%	0.0%	0.0%	2.2%	6.7%	4.4%	6.7%	4.4%	26.7%
Underrepresented/minorities	0.0%	0.0%	4.4%	4.4%	0.0%	2.2%	2.2%	2.2%	4.4%	4.4%	24.4%
Gold Humanism Honor Society membership	0.0%	0.0%	0.0%	2.2%	2.2%	2.2%	2.2%	8.9%	0.0%	4.4%	22.2%
Research Experience in Anesthesiology	0.0%	0.0%	0.0%	0.0%	2.2%	4.4%	4.4%	2.2%	0.0%	4.4%	17.8%
Other work experience	0.0%	0.0%	0.0%	2.2%	2.2%	2.2%	2.2%	2.2%	2.2%	2.2%	15.5%
Publications in Anesthesiology	0.0%	0.0%	2.2%	0.0%	0.0%	2.2%	0.0%	4.4%	2.2%	4.4%	15.5%
Publications in any field	0.0%	0.0%	0.0%	0.0%	0.0%	0.0%	0.0%	2.2%	6.7%	4.4%	13.3%
Community service	0.0%	0.0%	2.2%	2.2%	0.0%	0.0%	0.0%	0.0%	0.0%	4.4%	8.9%
Other (Please, specify)*	2.2%	2.2%	0.0%	0.0%	0.0%	0.0%	0.0%	0.0%	0.0%	2.2%	6.7%
Country of origin	0.0%	0.0%	0.0%	0.0%	0.0%	0.0%	0.0%	0.0%	0.0%	0.0%	0.0%
Work experience in the medical field	0.0%	0.0%	0.0%	0.0%	0.0%	0.0%	0.0%	0.0%	0.0%	0.0%	0.0%

Variability exists in how PDs rank factors when choosing who to interview. For example, of the PDs that had Step 1 in the top 10, 27% had it ranked between the 6th and 10th most important factor. 9% of PDs did not have Step 1 score in the top 10. 78% of PDs had letters of recommendation in the top 10, with 7% ranking letters #1 and 49% between the 6th and 10th most important. Core clinical clerkship grades were one of the top five factors by 49% of PDs, yet overall was the 6th most common top 10 factor as 36% of PDs did not have core clerkship grades at all in the top 10. 13% of PDs had country of medical education as the #1 factor (in aggregate country was 13th most commonly mentioned in the top 10).

Once Step 1 is reported as pass/fail, PDs had letters of recommendation (80%) as the most frequently ranked factor in the top 10, with 7% of PDs ranking it as the most important (Table 6). USMLE Step 2 CK (78%), and the MSPE (69%) were the next two most commonly ranked factors in the top 10.

**Table 6:** Ranking of importance of factors when selecting an applicant for an interview once Step 1 is reported as pass/fail Shows the importance of each factor in an application in order for the applicant to be invited for an interview, after the proposed change for the Step 1 score report to become pass/fail. *Local ties to the area, consistency in medical school performance, evidence of “grit”. MSPE = Medical School Performance Evaluation.

Factor	#1	#2	#3	#4	#5	#6	#7	#8	#9	#10	Total
Letter of recommendation	6.7%	15.6%	0.0%	4.4%	15.6%	8.9%	15.6%	6.7%	6.7%	0.0%	80.0%
USMLE Step 2 CK	31.1%	22.2%	8.9%	4.4%	0.0%	2.2%	0.0%	6.7%	0.0%	2.2%	77.8%
Dean's Letter (MSPE)	11.1%	8.9%	13.3%	6.7%	11.1%	6.7%	2.2%	4.4%	4.4%	0.0%	68.9%
Class rank	6.7%	6.7%	11.1%	13.3%	2.2%	13.3%	4.4%	2.2%	2.2%	2.2%	64.4%
Personal Statement	2.2%	0.0%	8.9%	4.4%	8.9%	4.4%	11.1%	11.1%	2.2%	8.9%	62.2%
Performance in preclinical courses	0.0%	2.2%	2.2%	6.7%	15.6%	6.7%	6.7%	2.2%	11.1%	6.7%	60.0%
Core clinical clerkship grades	8.9%	8.9%	8.9%	11.1%	2.2%	4.4%	0.0%	0.0%	4.4%	8.9%	57.8%
Leadership roles	0.0%	2.2%	2.2%	2.2%	4.4%	4.4%	6.7%	11.1%	6.7%	8.9%	48.9%
Research Experience in any field	0.0%	0.0%	2.2%	6.7%	0.0%	8.9%	8.9%	2.2%	4.4%	11.1%	44.4%
Grade in Anesthesiology clerkship	0.0%	2.2%	2.2%	8.9%	15.6%	0.0%	0.0%	4.4%	4.4%	6.7%	44.4%
Rotation at your department	6.7%	2.2%	4.4%	2.2%	2.2%	6.7%	0.0%	6.7%	4.4%	4.4%	40.0%
Applicant's medical school	2.2%	2.2%	8.9%	2.2%	0.0%	2.2%	8.9%	4.4%	4.4%	4.4%	40.0%
Written comments about clinical rotation performance	2.2%	4.4%	4.4%	6.7%	6.7%	4.4%	2.2%	2.2%	4.4%	2.2%	40.0%
Alpha Omega Alpha Honor Medical Society	4.4%	6.7%	0.0%	4.4%	0.0%	0.0%	2.2%	8.9%	2.2%	4.4%	33.3%
Research Experience in Anesthesiology	0.0%	0.0%	6.7%	0.0%	4.4%	4.4%	0.0%	4.4%	6.7%	2.2%	28.9%
The number of Anesthesiology electives	0.0%	0.0%	0.0%	2.2%	2.2%	2.2%	8.9%	2.2%	2.2%	6.7%	26.6%
Publications in any field	0.0%	0.0%	0.0%	2.2%	2.2%	4.4%	0.0%	2.2%	8.9%	2.2%	22.2%
Country of medical education	11.1%	4.4%	2.2%	0.0%	0.0%	0.0%	2.2%	2.2%	0.0%	0.0%	22.2%
USMLE step 1 pass/fail	0.0%	4.4%	4.4%	0.0%	0.0%	4.4%	0.0%	0.0%	4.4%	4.4%	22.2%
Publications in Anesthesiology	2.2%	0.0%	2.2%	0.0%	2.2%	0.0%	4.4%	4.4%	2.2%	4.4%	22.2%
Gold Humanism Honor Society membership	0.0%	2.2%	0.0%	0.0%	2.2%	4.4%	4.4%	4.4%	2.2%	2.2%	22.2%
Underrepresented/minorities	0.0%	2.2%	0.0%	6.7%	0.0%	0.0%	2.2%	2.2%	4.4%	0.0%	17.8%
Other work experience	0.0%	0.0%	2.2%	2.2%	2.2%	4.4%	4.4%	0.0%	0.0%	0.0%	15.5%
Community service	0.0%	0.0%	2.2%	0.0%	0.0%	0.0%	2.2%	2.2%	4.4%	2.2%	13.3%
Other (Please, specify)*	4.4%	2.2%	0.0%	0.0%	0.0%	0.0%	0.0%	2.2%	0.0%	0.0%	8.9%
Work experience in the medical field	0.0%	0.0%	0.0%	0.0%	0.0%	0.0%	0.0%	0.0%	2.2%	0.0%	2.2%
Country of origin	0.0%	0.0%	0.0%	0.0%	0.0%	0.0%	0.0%	0.0%	0.0%	0.0%	0.0%

With regard to characteristics that may indicate an applicant may be less interested in actual training in their program, not having ties to their geographic region was the most commonly written in response by PDs. Examples of such verbatim comments included: “If not from the Midwest, I assume they really don't want to relocate to my area,” “Lack of any connection to the region.” Other factors mentioned that an applicant may not desire to train at a program included someone doing (basic) research in a field not strong/offered by their institution, research-oriented people who look for a safety program, and couples match applicants.

Restricting the number of programs a candidate can apply to was supported by 64% of the respondents. The majority suggested the number should be limited to 15 to 20 programs per applicant.

## Discussion

Even though Step 1 may be a source of anxiety, financial burden [10,11], and life crisis [12] for students, most of the 45 anesthesiology PD respondents disagreed with changing the Step 1 score report from a 3-digit score to pass/fail. Since 84% of PDs consider the Step 1 score as moderately or very important for selecting an applicant for interview, changing the Step 1 to pass/fail will modify how programs evaluate applicants for interview. Likely, the Step 2 Clinical Knowledge (CK) score will increase in importance. At the time the survey was conducted, the Step 2 CK score was ranked in the top 10 by 69% of PDs, and was ranked in the top 10 by 78% of PDs once Step 1 is pass/fail.

Noteworthy variability exists in how PDs perceive candidate factors when selecting an applicant for an interview. For example, of the PDs that had Step 1 score in the top 10, 27% had it ranked between the 6th and 10th most important factor, and 9% did not have it in the top 10 at all. This suggests that many programs do not currently utilize Step 1 score for interview decisions. Once Step 1 becomes pass/fail, the Step 2 rises in importance, with 31% ranked it as the single most important, and 22% as second most important. Yet with Step 1 as pass/fail, 22% of PDs indicated the Step 2 score would not be one of the top 10 factors of importance. Core clinical clerkship grades were listed as one of the top 5 factors by 49% of PDs suggesting that half of the PDs view it as a crucial metric, yet overall was the 6th most important because approximately 40% of PDs did not have it in the top 10 when choosing who to interview. 

USMLE Step 2 CK score is a measure of an applicant's ability to apply the medical knowledge, skills, and understanding of clinical science essential for providing patient care. If many programs across specialties do place more importance on Step 2 once Step 1 is pass/fail, then the risk is creating a new “Step 2 Culture,” whereby students may again feel the burden of preparing for that examination and medical schools may shift curriculum to teaching to the test.

Once the Step 1 three-digit score is not available, the PDs indicated that letters of recommendation will move from second most important to first most important in aggregate to make interview decisions. Letters came out as # 1 overall in part because most (4 out of 5) PDs had letters listed somewhere in the top 10, with “content of the letter” and “how well the letter writer knows the applicant” as key parts of the letter.

Once Step 1 scores are pass/fail, PDs may also place more value on research experience (moved from 17th to 9th position in top 10 importance). PDs identified “any research experience” (23%) and “research experience but not necessarily publication” (20%) as important. U.S. seniors averaged 3.3 research experiences (e.g., abstracts, presentations, and, publications) with 85% reporting this information, and those who matched had more research experiences on average [13]. It can be difficult to properly evaluate the quality of the research experience by simply looking at what’s available in the application. Pre-residency scholarship productivity is a predictor of post-residency career academic productivity [14].

Once Step 1 scores are pass/fail, PDs indicated that performance in preclinical courses (moved from 15th to 6th position) will be more important even though most medical schools have moved to pass/fail only for preclinical courses [15]. The personal statement (moved from 7th to 5th position) will increase in importance once Step 1 is reported as pass/fail. It may be that some programs will transition away from test scores and course grades and move towards a holistic application review that includes an increased emphasis on the personal statement. This may cause an increase in the amount of work programs will have when looking at applications, having to spend more time than "simply" looking at a 3-digit score.

One unintended consequence of not having a three-digit Step 1 score is that applicants from lesser-known medical schools may have less opportunity to interview at elite programs because they don’t have a high Step 1 score. As a result, holistic review becomes imperative with PDs considering the “whole” applicant and their experiences, attributes, and academic metrics, rather than overemphasizing any one factor [16]. Holistic admissions are mission-based, and therefore the selection criteria and outcomes will vary by institution. Residency programs may place emphasis on other aspects of the application such as interpersonal attributes, motivation for anesthesia, diversity, community service, and overcoming disadvantages. Holistic reviews have been used in medical, nursing and dental schools to increase the number of underrepresented minorities, or to target the skills, attributes, and behaviors sought in future health care providers [10].

Residency programs are more likely to do a holistic review once an applicant has been interviewed to determine where the applicant should be ranked in the match. Holistic reviews for interview invitation decisions are difficult because of the time required. For example, if a program receives 1000 applications, and 30 minutes are spent on each application, then the PD would need to work full-time (40 hours/week) for three months to review all the applications [17]. PDs could give individualized consideration to how each applicant may contribute to their specific learning environment and the outcomes desired by the institution’s mission, vision, and values [18]. Automatically screening out an applicant who has been arrested or convicted may be problematic because it presents a risk of unfairness to applicants of color [19].

Screening applications for interview invitations also involves the use of “red flags” or items that raise concerns that may eliminate the opportunity to interview. The most frequently mentioned red flags were failure of a USMLE exam, failure of a course or clinical rotation, gaps in education/missing time from school without explanation, a criminal history, professionalism issues/negative comments, and lukewarm/poor/short/generic letters of recommendation. Another factor diminishing a candidate’s chances to interview at a program was if the applicant lacked any relationship to the residency’s institution or area of the country. In contrast, most PDs gave priority to applicants from the medical school affiliated with their residency program. An applicant living in the same state as the interviewing program is more likely to match there than an out of state applicant [20].

As the number of programs each US medical student applies to increases each year (60 in 2018), PDs may not be able to complete holistic reviews [21]. Restricting the numbers of programs a candidate can apply to was supported by 64% of PDs, with the majority suggesting a limit of 15 to 20 programs per applicant. Although this may benefit residency programs, students would have to be more targeted in seeking a residency, thereby needing to be adequately counseled as to which programs they would be competitive for or a “good fit.”

Efficient, unbiased methods for screening residency candidates deserve further study. A software algorithm for an otolaryngology residency that automatically scored components of each student's Electronic Residency Application Service application using predetermined criteria decreased the time needed to review applications without impacting the composition of the interviewee pool [22].

Once the candidate is invited for an interview Situational Judgment Tests can be used to try to predict success at a program. Situational Judgment Tests are based on hypothetical scenarios and assess how one approaches situations encountered in the workplace, analyzing how the candidate would react to each situation. Performance on such tests are associated with higher resident performance, and may be better to predict professionalism than alternative screening approaches [23,24].

One limitation of this study is that the survey was not designed to address osteopathic applicants. These applicants have the Comprehensive Osteopathic Medical Licensing Examination of the United States (COMLEX-USA) as the most common pathway to apply for medical licensure in a State. Unlike Step 1 the COMLEX Level 1 will continue to have a three-digit score. Osteopathic students often take Step 1 to provide a direct comparative measure for allopathic applicants and PDs. When Step 1 becomes pass/fail, osteopathic students may feel the need to take Step 2.

It is not clear that educational value will increase by creating a holistic learning environment for medical students once the Step 1 score moves to pass/fail. This could occur for example if other indicators influence students in unanticipated ways, such as the subjective (i.e. recommendation letter) and negative (i.e. red flags experiences) factors left for resident selection. If the overall goal is to optimize educational purpose and teaching and learning experience, the potential of unintended consequences of not having a Step 1 score was not specifically a goal of this survey study. 

Other limitations of the study include that the questionnaire instrument was developed specifically for this study without formal psychometric testing. Our methodology was based on the ranking analysis of the top 10 factors, which treats the top 10 rankings as equal, without giving weight to higher-ranked factors, which may have changed the final rank order of factors. Face and content validity were addressed by repeated group discussions among the study authors and pilot testing with iterative improvements to the survey. Another limitation is the response rate, and corresponding response bias. Although respondents were distributed in all four major geographic areas, and with different types of institutions responding, this may not correctly reflect a national perspective. Also, these results are from anesthesiology PDs and may not reflect other specialties. With regard to how PDs ranked factors (e.g., some programs don’t consider Step 1 at all, some programs ranked country of origin as most important), this study’s goal was not to assess if the type of program (small versus large, university versus community) impacts how the factors are ranked.

## Conclusions

In conclusion, Step 1 will become pass/fail in 2022 in part because the score of a single exam shouldn’t dominate medical students’ precious time and learning environment. Although there is variability among anesthesiology PDs in their criteria for offering interview, there will be an increased emphasis on Step 2 CK scores once Step 1 is reported as pass/fail.

## Appendices

Survey

1.       What region is your program located?

(  ) Midwest       (  ) South             (  ) West Coast                  (  ) East Coast

2.       How is your program classified?

(  ) Community hospital                (  ) University Hospital

(  ) University Affiliated                 (  ) Military Program

(  ) Other (Please, specify)

3.       How likely are you to agree with the proposed change made to the Step 1 score report?

(  ) Strongly disagree      (  ) Disagree       (  ) Agree             (  ) Strongly agree

4.       How important is the Step 1 score for selecting an applicant for interview?

(  ) Not at all important                 (  ) Slightly important

(  ) Moderately important            (  ) Very important

5.       Currently, when selecting applicants to interview, which aspects are most important? Please rank the top 10 by dragging the options, 1 being the most important and 10 the least important.

(  ) Research experience in Anesthesiology                 (  ) Other work experience

(  ) Publications in Anesthesiology                                  (  ) Publications in any field

(  ) Country of origin                                                              (  ) Community service

(  ) Work experience in the medical field                      (  ) Dean’s Letter (MSPE)

(  ) USMLE Step 1                                                                    (  ) Letter of Recommendation

(  ) USMLE Step 2 CK                                                              (  ) Core clinical clerkship grades

(  ) Alpha Omega Alpha Honor Medical Society          (  ) Research Experience in any field

(  ) Personal Statement                                                        (  ) Class rank

(  ) Applicant’s medical school                                           (  ) Leadership roles

(  ) Grade in Anesthesiology clerkship                            (  ) Rotation at your department

(  ) Written comments about clinical rotation performance

(  ) Performance in preclinical courses                           (  ) Country of medical education

(  ) The number of Anesthesiology electives                (  ) Underrepresented/minorities

(  ) Gold Humanism Honor Society membership

(  ) Other (please, specify)

6.       A “red flag” is any aspect of the applicant profile that may raise concerns about the qualities of the applicant. What red flags do you consider when reviewing an application? Please leave a comment.

7.       How important are red flags when selecting an applicant for interview?

(  ) Not at all important                 (  ) Slightly important

(  ) Moderately important            (  ) Very important

8.       Please, select the two most important characteristics of a Letter of Recommendation:

(  ) Content of the letter               (  ) How well the letter writer knows the applicant

(  ) Specificity of the letter           (  ) From someone in the specialty

(  ) From someone you know     (  ) From a well-recognized person

(  ) Other

9.       Please, select the two most important characteristics in Research Experience and Publications.

(  ) Quantity of publications         (  ) Basich science research

(  ) Any research experience       (  ) Research in Anesthesiology

(  ) Research experience but not necessarily publication

(  ) Quality of publication (high impact factor)

(  ) Applicant is middle author    (  ) Applicant is first author

(  ) Other

10.   An applicant from the Medical School of your program has advantage over other applicants.

(  ) Strongly disagree      (  ) Disagree        (  ) Agree             (  ) Strongly agree

11.   How important is it for the applicant to be from the State/Area of your program?

(  ) Not at all important                 (  ) Slightly important

(  ) Moderately important            (  ) Very important

12.   How important is Step 2 Clinical Knowledge (Step 2 CK) score for selecting an applicant for an interview?

(  ) Not at all important                 (  ) Slightly important

(  ) Moderately important            (  ) Very important

13.   The Step 1 score report will chance from a three-digit score to only a pass/fail outcome. When this occurs, which aspects will be most important when selecting applicants for an interview? Please rank the top 10 by dragging the options, 1 being the most important and 10 the least important.

(  ) Country of Origin                                      (  ) Work experience in the medical field

(  ) Community service                                  (  ) Other work experience

(  ) Underrepresented/minorities            (  ) Publications in Anesthesiology

(  ) USMLE Step 1 pass/fail                           (  ) Gold Humanism Honor Society membership

(  ) USMLE Step 2 CK                                      (  ) Letter of Recommendation

(  ) Dean’s Letter (MSPE)                              (  ) Personal Statement

(  ) Class rank                                                    (  ) Core clinical clerkship grades

(  ) Performance in preclinical courses    (  ) Leadership roles

(  ) Research experience in any field        (  ) Grade in Anesthesiology clerkship

(  ) Rotation at your department              (  ) Applicant’s medical school

(  ) Written comments about clinical rotation performance

(  ) Alpha Omega Alpha Honor medical Society

(  ) Research experience in Anesthesiology         

(  ) The number of Anesthesiology electives

(  ) Publications in any field                         (  ) Country of medical education

(  ) Other (Please, specify)

14.   Are there any characteristics you look for in an application that may indicate that the applicant is less likely to be interested in training in your program and therefore less likely for you to invite for an interview?

15.   Do you think a candidate should have a restricted number of programs he/she could apply for?

(  ) yes  (please, specify how many programs)

(  ) no

16.   If you have any additional information you would like to share on how you intend to overcome this change, please include them below.
